# Chronic Study on Brainwave Authentication in a Real-Life Setting: An LSTM-Based Bagging Approach

**DOI:** 10.3390/bios11100404

**Published:** 2021-10-18

**Authors:** Liuyin Yang, Arno Libert, Marc M. Van Hulle

**Affiliations:** Department of Neuropsychology and Physiology, KU Leuven, 3000 Leuven, Belgium; arno.libert@kuleuven.be

**Keywords:** EEG-based authentication, multitask authentication, real-world setting, EEG headset

## Abstract

With the advent of the digital age, concern about how to secure authorized access to sensitive data is increasing. Besides traditional authentication methods, there is an interest in biometric traits such as fingerprints, the iris, facial characteristics, and, recently, brainwaves, primarily based on electroencephalography (EEG). Current work on EEG-based authentication focuses on acute recordings in laboratory settings using high-end equipment, typically equipped with 64 channels and operating at a high sampling rate. In this work, we validated the feasibility of EEG-based authentication in a real-world, out-of-laboratory setting using a commercial dry-electrode EEG headset and chronic recordings on a population of 15 healthy people. We used an LSTM-based network with bootstrap aggregating (bagging) to decode our recordings in response to a multitask scheme consisting of performed and imagined motor tasks, and showed that it improved the performance of the standard LSTM approach. We achieved an authentication accuracy, false acceptance rate (FAR), and false rejection rate (FRR) of 92.6%, 2.5%, and 5.0% for the performed motor task; 92.5%, 2.6%, and 4.9% for the imagined motor task; and 93.0%, 1.9%, and 5.1% for the combined tasks, respectively. We recommend the proposed method for time- and data-limited scenarios.

## 1. Introduction

Authentication is the process of verifying a claimed identity, a vital aspect of information security [1]. Contrary to identification, where the system identifies the individual’s identity based on information provided, such as name, user name, and user ID, authentication verifies the claimed identity [1]. Traditionally, authentication methods require users to provide personal information, such as passwords or specific phrases, or, alternatively, smart tokens or credit cards. According to Menkus, these methods refer to “knowledge” and “possession”, respectively [2]. Although widely used, these authentication methods are vulnerable to shoulder surfing, forgery, and theft [3]. Biometric-based authentication is witnessing growing interest, as it takes advantage of biometric traits that are highly correlated with the individual, leading to a higher degree of security [2]. Biometric traits used in daily-life settings are fingerprints, the iris, and facial characteristics, albeit some are not without concerns. Some biometrics have been proved to be discriminatory towards certain groups such as individuals with physical disabilities, as they may fail to provide them [4]. Electroencephalogram (EEG), on the other hand, avoids such caveats while, at the same time, adding some unique features, e.g., by tapping into the neural features of the user [4], creating a countermeasure to external pressure [5]. A variety of EEG-authentication paradigms have been proposed, among which are resting-state, i.e., resting with the eyes open or closed, analysis [6]; performed or imagined limb or tongue motor activity [7,8,9]; visual evoked potentials (VEPs); and mental tasks such as self-face image recognition [10], mental counting, and geometric figure rotation [11].

Recently, Barayeu et al. [7] reported an accuracy of 95.64%, and an FAR of 1.26% when using 64-channel EEG recordings in response to a performed motor task and a PCA-SVM decoder. Chatterjee et al. [8] applied ensemble learning techniques to decode EEG in response to an imagined motor task using various weak classifiers operating on wavelet energy, entropy, and band power features, and adaptive autoregressive parameters. The highest accuracy they achieved was 85.71% with a mix-boost ensemble classifier based on band power, which performed better than non-ensemble methods. Unfortunately, the FAR and FRR were not reported. Das et al. [9] applied a convolutional neural network (CNN) for automatic discriminative feature extraction and authentication using a motor imagery task, yielding an accuracy of up to 99.3%; the FAR and FRR were not reported. Zeng et al. observed a significant improvement after combining the rapid serial visual presentation (RSVP) of faces paradigm with eye blinking signal features [12]. They used a CNN for score estimation and reported an average accuracy of 97.6%, FAR of 2.71%, and FRR of 2.09%, demonstrating the advantage of multitask authentication. Sun et al. combined CNN with a 1D long short-term memory (LSTM) network [13], yielding an accuracy of 97%, an FAR of 0.03, and an FRR of 0.03, which was higher than that of CNN or LSTM individually. In addition, some experiments that have demonstrated the feasibility of EEG-based mobile phone authentication have been conducted [14]. In this study, we evaluated, for the first time, an EEG-based form of authentication in a real-world setting. EEG data were recorded over several days using a commercial eight-channel dry electrode EEG headset (Unicorn Hybrid Black [15]) in a non-laboratory environment. We proposed a novel LSTM-based bootstrap aggregating (bagging) decoder to authenticate the user of the device. In contrast to [7,8,9], where multiple, complex features were extracted, the proposed method relies on spectral information only, leading to an efficient training process. The network architecture we propose took, on average, 0.135 s (std., 0.004), 16.59 s (std., 1.290), and 0.106 s (std., 0.028) to load, train one ensemble, and classify from nine ensembles, respectively. We compared the authentication performance under different resource uses given non-ensemble- and ensemble-based learning. We show that the latter is more suited for a real-life setting, given limited time and data resources, yet providing good scalability.

## 2. Materials and Methods

### 2.1. Ethics

Prior to the experiments, our participants read and, when they agreed, signed the consent form previously approved by the ethical committee of KU Leuven’s university hospital (UZ Leuven) under registry number B322201940585 (communication code: S62547), approved on 7 June 2019.

### 2.2. Experimental Setup

**Figure 1:**
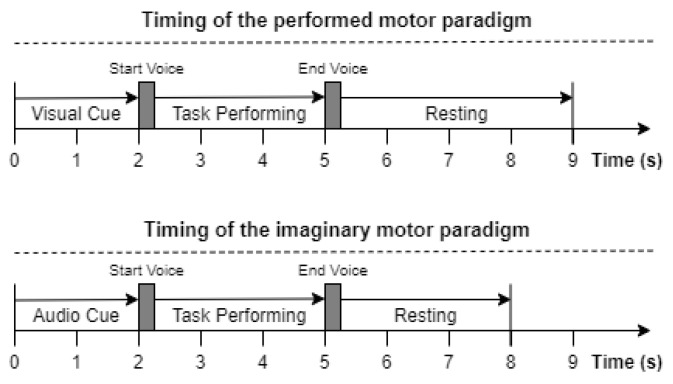
Timing of the tasks, showing resting period and instruction, start, and end cues (see text).

We recruited 15 healthy subjects (9 males and 6 females; age range: 18–28 years, with a mean of 24.1 and a standard deviation of 3.2). None of the participants suffered from any neurological disease or were on any medication. Each subject participated in at least 5 sessions at a rate of one per day. The sessions were either on consecutive days or with several days in between. To simulate a real-world setting, the sessions took place in the subject’s residence, except for three subjects, whose sessions were conducted in the lab (Participants 1, 2, and 3). We minimized external interferences by ensuring all the lights were switched off, as well as electronic devices; smartphones and laptops, except the recording laptop, were in flight mode and kept at a distance from the EEG device. Throughout each session, the subjects sat in a comfortable position and were asked to follow our instructions presented on the screen. Ten trials of the performed motor paradigm were first conducted, of which 5 were with the left and 5 the right arm, all in a random order, followed by 10 trials of the imagined motor paradigm (see below for further detail). In between the performed motor and the imagined motor paradigms, a clear instruction was given on the screen. The timing of the performed and imagined motor paradigms is shown in Figure 1.

**Figure 2:**
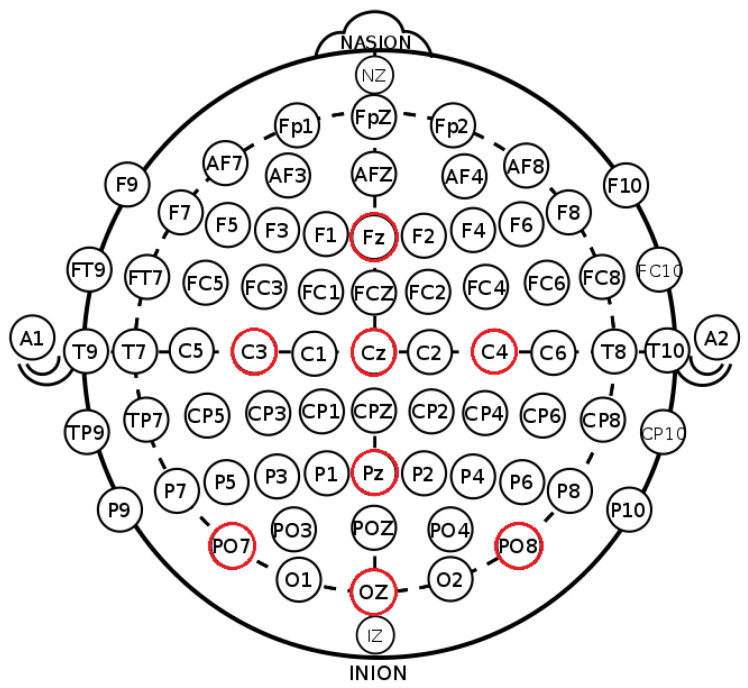
Location of the used EEG electrodes (marked in red): Fz, C3, Cz, C4, Pz, PO7, Oz, and PO8, according to the international 10–20 system.

In the performed motor paradigm, a visual cue was presented on the screen of the recording laptop, indicating which arm to raise. After 2 s, a “start” instruction was voiced, indicating the start of the cued task. The subject had 3 s to perform the task, after which an “end” instruction was voiced, indicating the end of the task. Each trial was followed by a resting period of 4 s before the onset of the next trial. In the imagined motor paradigm, the subjects were instructed to close their eyes and listen to an audio instruction cue. When the “start” cue was played, they were asked to imagine raising the left or right arm in accordance with the instruction cue. After 3 s of movement imagination, the “end” cue was played, followed by a 3-second resting period. EEG data were recorded using the Unicorn Hybrid Black headset (https://www.unicorn-bi.com/ accessed on 20 September 2020), a portable, wireless dry-electrode EEG headset equipped with 8 recording electrodes, operating at a 250 Hz sampling rate. The electrode locations were as shown in Figure 2. All the recording electrodes were referenced to the mastoids (linked mastoid referencing).

### 2.3. Preprocessing and Feature Extraction

The raw data from the 8 electrodes were filtered using a fourth-order Butterworth bandpass filter with cutoff frequencies at 1 and 30 Hz. Subsequently, the data were downsampled to 100 Hz and cut into epochs corresponding to the 3-s task interval (as shown in Figure 1). The results of each trial consisted of 8×300 time samples representing EEG recordings from 8 electrodes in the 3-s period. In accordance with [16], we took 7–12 Hz as the mu band and 12–30 Hz as the beta band, the two major frequency bands modulated during motor tasks [17]. The short-time Fourier transform (STFT) was used to compute the spectrogram of each electrode signal, serving as input to the long short-term memory networks (LSTMs) (see below for further details). We applied the short-time Fourier transform (STFT) as follows. The 300-point sampled amplitudes were divided into 128-point segments, with 98% overlap between two consecutive segments. Each segment was tapered by a Hanning window [18] to suppress the leakage of low-frequency components [19]. Then, the 128-point STFT was computed for each windowed segment, resulting in a 65×68 matrix. Each row corresponds to a frequency component with a resolution of 0.78 Hz, ranging from 0 to 50 Hz (i.e., half of the sampling frequency), while each column represents a 44 ms interval. Next, the mu (7–12 Hz) and beta (12–30 Hz) bands were selected and grouped into 5 sub-bands (7–11 Hz, 11–16 Hz, 16–21 Hz, 21–25 Hz, and 25–30 Hz). The corresponding power amplitudes were calculated as follows:

(1) $$P_{xf}=20*log_{10}\left|X_{f}\right|,$$

where $P_{xf}$ is the power expressed in dB for the frequency band *f* and $X_{f}$ is the complex STFT spectrum of the frequency band *f*, leading to a 5×68 power spectral matrix with information from the above 5 sub-bands, for each channel. The feature matrix was constructed by concatenating the 5 power spectral matrices row-wise, resulting in a 25×68 matrix.

### 2.4. LSTM-Based Recurrent Neural Network

**Figure 3:**
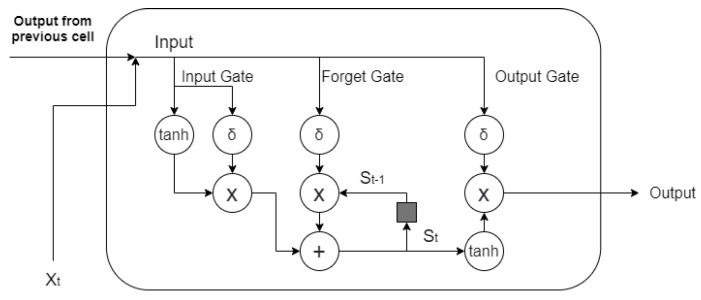
LSTM cell structure, adapted from [22].

Long short-term memory (LSTM) networks are a type of recurrent neural network (RNN) dedicated to learning from time-sequence data [20]. When dealing with long sequences, as the RNN suffers from the vanishing gradient problem [21], LSTM was designed to solve this problem with its special gate cell structure. The gate cell structure enables the LSTM to memorize important information from past data whilst keeping track of more recent information. As shown in Figure 3, the cell decides when to allow reads, writes, and erasures via different gates: concatenated with the latest arrival data $X_{t}$, the input data pass through the input gate via a gate activation function δ, multiplied by the output from a tanh layer, representing which values to update, and new candidate values at this stage. The new values are added to the previous values, scaled by forget factors through the gate activation function δ, multiplied with the previous calculated features $S_{t-1}$. Finally, an output is calculated through the tanh function multiplied with weights calculated from the output gate activation function δ.

**Figure 4:**
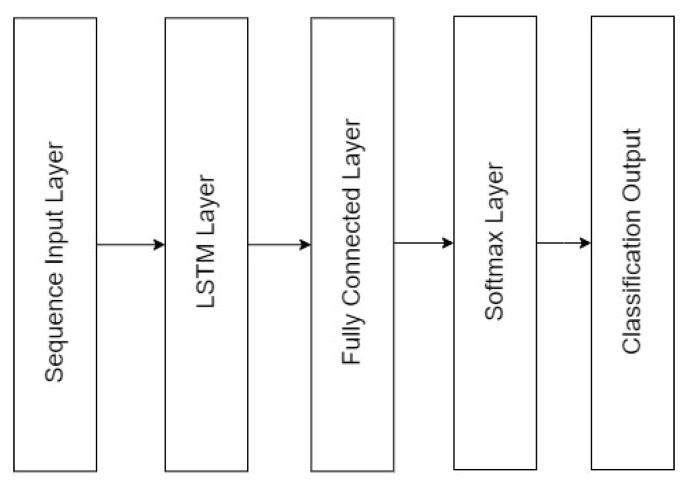
Neural network structure used for EEG-based authentication.

In [23], the authors successfully applied an LSTM-based network with visual stimuli and achieved an average accuracy of 91.44%. This inspired us to adopt this architecture for our application (Figure 4), with the spectral features passing through an LSTM layer as sequence data, with the output neurons fully connected, and a prediction using the Softmax function. This shallow network was used as a weak learner in the ensemble technique discussed later.

### 2.5. Bagging

In a real-life EEG-based authentication setting, the size of the training data is always going to be restricted, as the user is not expected to participate in lengthy data-collection sessions, which is an additional challenge compared to other biometric traits such as fingerprints, where data collection only takes a few seconds. To address this concern, we considered a limited dataset, but with the additional complication that the recordings were originating from different days, potentially with slightly different EEG-electrode locations and signal qualities. To account for such limited and variable datasets, we used bagging (bootstrap aggregation), an ensemble technique capable of substantially improving decoding accuracy [24]. The authentication model was trained on a bootstrap sample with the same number of data points but randomly drawn from the original data set (with replacement) (Algorithm 1). The ensemble size (bagging size) was defined as M. Given the M model predictions, the majority vote decided on the final outcome (i.e., to authenticate or not).
**Algorithm 1:** Bagging.
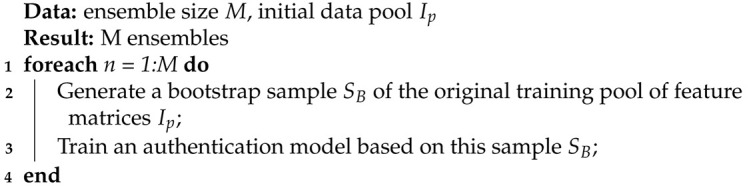


### 2.6. Authentication

We considered two authentication scenarios serving different objectives. In the first scenario, one authentication model was developed per subject considering the remaining 14 (=15−1) subjects as imposters. Hence, the data of all 15 subjects were used for training the models, and their purpose was to assess their accuracy in predicting authentication (classifier output 1 = Yes; 0 = No) over time (cf. chronic use). In the second scenario, we divided our 15 subjects into two groups: 10 vs. 5. We developed one authentication model per subject using the data of the nine remaining subjects of the first group as imposters; subjects from the second group (five subjects) were considered imposters as well, but their data were not used for training the authentication model. This scenario was intended to test the case where unknown individuals attempted to obtain access.

**Figure 5:**
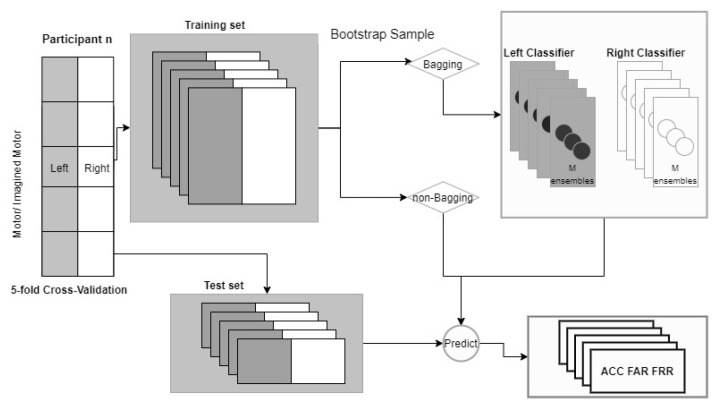
Overall workflow of the bagging approach.

Figure 5 illustrates the workflow of the proposed method. For both scenarios, as we targeted chronic use, we used, as a training set, the data from all five recording days, expect 1 day, for which we used the data as the test set in a 5-fold cross-validation setting, with the left and right arm trials kept separate. The aggregated training set was used for the standard LSTM network without and with bagging, where the “Yes” training samples were drawn from the corresponding subject and the “No” training samples, from all the other users (14 in the first and nine in the second authentication scenario). To avoid a biased training set, the number of negative samples taken from unauthorized users was equal to the positive samples taken from the authorized user. Each bootstrap sample was used to train an LSTM-based neural network. After training, each subject had a left and a right arm classifier, for each of which M models were trained. Ideally, the classifier predicts a “Yes” for the EEG trial of the targeted subject and a “No” for all the other subjects (“imposters”). We collected performance statistics in terms of accuracy, FAR, and FRR. To compare the LSTM approach with and without bagging, we examined the effect of varying the training size, where LSTM models without bagging were trained using a small subset containing only between 10% and 90% of the aggregated training set; bagging was applied on the same subset. The results were compared on the same test set. To reduce the influence of random factors, several runs were conducted, and the averages were calculated and are reported.

### 2.7. Statistics

A pairwise Wilcoxon sign-rank test was used to investigate the significance between the accuracy, FAR, and FRR for the various authentication paradigms. The zero hypothesis was set to be that both distributions were drawn from a distribution with a common mean. The significance threshold was set at α=0.05.

## 3. Results

**Figure 6:**
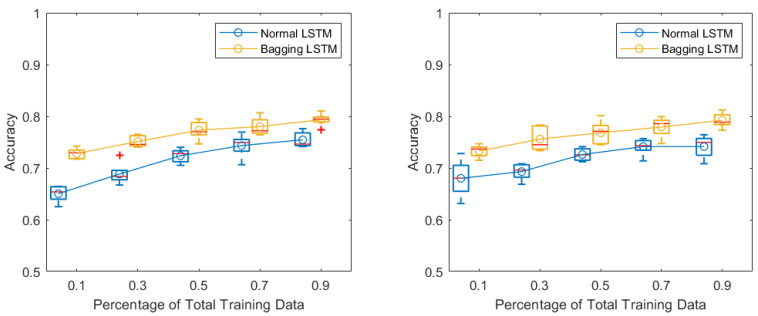
(**left**) Accuracy of standard LSTM vs. bagging LSTM in the performed motor task. (**right**) Accuracy of standard LSTM vs. bagging LSTM in the imagined motor task, plotted as a function of the percentage of total training data. Boxplots of 5-fold cross-validation results with medians marked by red bars and boxes referring to 1st and 3rd quartiles. The curves connect the means, marked by circles.

First, we studied the performance of the standard LSTM network and the one using the bagging approach with an ensemble size of M = 5. The size of the training set varied between 10% and 90% of the data in the aggregated training pool. The boxplots corresponding to the 5-fold cross-validation results of the performed and imagined motor tasks are presented in Figure 6.

An increasing trend for the accuracy can be observed from the graph. When 10% of the training set was used for the actual training (six positive and six negative classes), the bagging approach achieved accuracies higher than 0.70 on both the performed and imagined motor tasks, while the standard LSTM approach achieved an accuracy only around 0.65. When 90% of the data were used for training, both approaches achieved accuracies around 0.80. A significant difference between the two methods, over all the training sizes, can be observed (Wilcoxon signed-rank test results for the performed motor and imagined motor tasks: $1.2290\times 10^{-5}$ and $1.2279\times 10^{-5}$).

**Figure 7:**
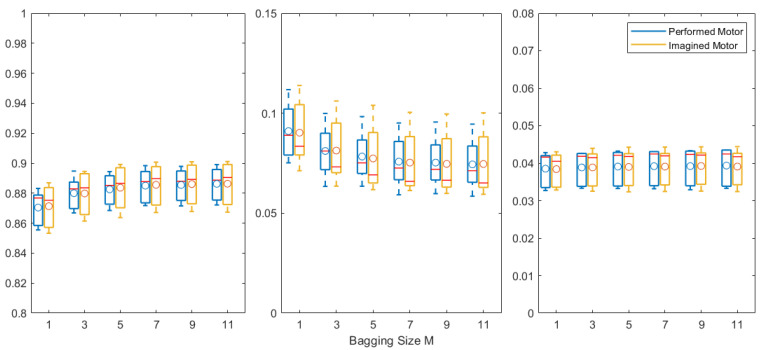
Boxplots of (**left**) accuracy, (**center**) FAR, and (**right**) FRR for performed and imagined motor tasks, plotted as a function of bagging size M. Other conventions are as in Figure 6.

The accuracy, FAR, and FRR obtained from different bagging sizes are presented in Figure 7 (5-fold cross-validation results); when the ensemble size increased, no significant improvement was observed except for bagging size M = 1, for which the accuracy was the lowest and the FAR was the highest. The performed motor results showed a similar distribution over the subjects to that of the imagined motor results. When using M = 9 bagged models, we obtained, for the performed motor task, an average accuracy of 0.8855, FAR of 0.0753, and FRR of 0.0392, and 0.8861, 0.0747, and 0.0393 for the imagined motor task, respectively.

**Figure 8:**
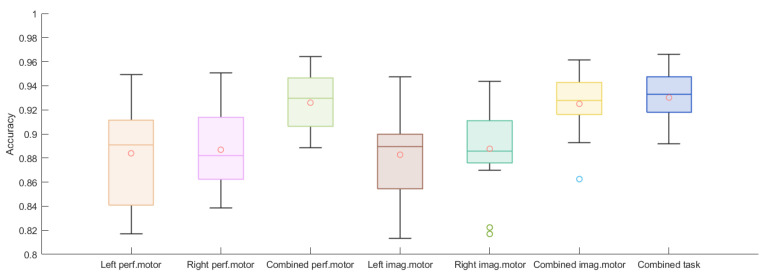
Boxplots of 15 subjects’ averaged accuracies for 5-fold cross-validation for left performed motor, right performed motor, combined performed motor, left imagined motor, right imagined motor, combined imagined motor, and combined tasks.

**Figure 9:**
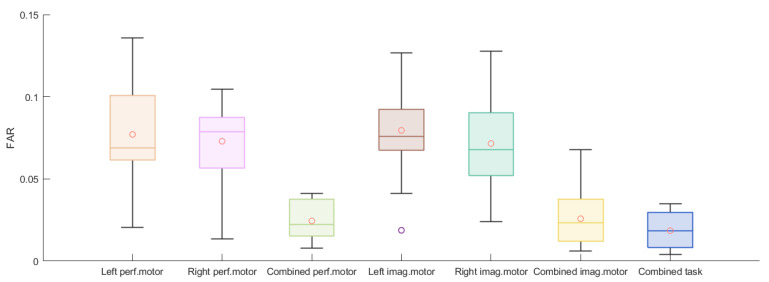
Boxplots of 15 subjects’ averaged FAR for 5-fold cross-validation for left performed motor, right performed motor, combined performed motor, left imagined motor, right imagined motor, combined imagined motor, and combined tasks.

**Figure 10:**
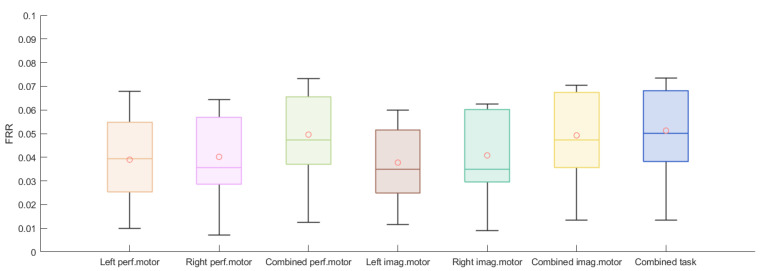
Boxplots of 15 subjects’ averaged FRR for 5-fold cross-validation for left performed motor, right performed motor, combined performed motor, left imagined motor, right imagined motor, combined imagined motor, and combined tasks.

The accuracy, FAR, and FRR of each authentication scheme for the M = 9 bagged models are listed in Figure 8, Figure 9 and Figure 10. The individual results are reported in Supplementary Tables S1–S3. For the performed motor tasks, the highest accuracy was 0.964 and the lowest, 0.897. The highest accuracy for the imagined motor task was 0.962; the lowest, 0.893. For both tasks, for most of the participants, the FARs were smaller than 0.08 and the FRRs were smaller than 0.05. Furthermore, when the data from the left and right arm tasks were combined, the average accuracies improved and, at the same time, the FAR dropped while the FRR increased. A Wilcoxon signed-rank test was applied to assess whether the differences in performance between the paradigms were significant. The results are listed in Supplementary Tables S4–S6 and reveal a significant increase in accuracy for the combined paradigms compared to the single left and single right paradigms for the performed and imaginary motor tasks. Additionally, combining all the tasks significantly outperformed combining only the performed and imaginary motor tasks. A similar observation can be made for the FAR. The combined tasks had a significantly higher FRR compared to the single tasks, and it was the worst when combining all the tasks.

Moreover, Figure 8 shows that the accuracies from the two-task authentication scheme (when the performed motor task was combined with the imagined one) had no apparent improvement over the single-task accuracies, whilst we observe a dramatic decrease in the FAR from Figure 9, as nearly no false acceptance was observed (smaller than 0.02 for most of the subjects), whereas the FRR increased as shown in Figure 10. The two-task authentication had a minimum accuracy of 89.1% and a maximum FRR of 7.3%. Note that the results of the three participants whose data were collected in the lab (Participants 1, 2, and 3) were no different from those of the others, indicating the feasibility of EEG-based authentication in a real-world setting. Table 1 shows the average results for the different tasks. In general, all the tasks achieved above 85% accuracy and the performed motor task exhibited the highest accuracy and lowest FRR. On the other hand, the two-task scheme had the lowest FAR and highest FRR.

**Figure 11:**
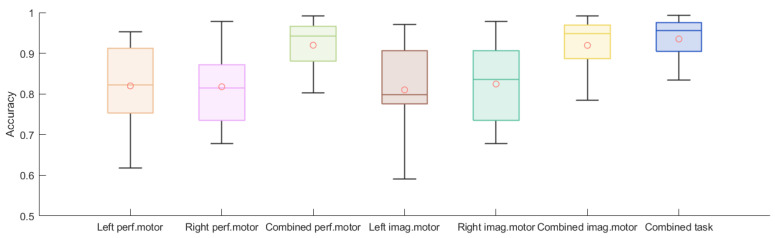
Boxplots of 15 subjects’ accuracies averaged over 5 imposters for left performed motor, right performed motor, combined performed motor, left imagined motor, right imagined motor, combined imagined motor, and combined tasks.

Figure 11 shows the accuracies of the trained models successfully rejecting authentication attempts from imposters unknown to the system. Each authentication scheme achieved an average accuracy above 0.8. The combined performed motor scheme and the combined imagined motor scheme had higher means as well as lower variances than the non-combined schemes. The combined task scheme achieved the best performance, with the highest mean value (93.0%) and the smallest variance (0.0005).

Finally, it took 0.135 s (std., 0.004) to load one model (consisting of nine bagged LSTMs). Training one LSTM-based ensemble took 16.59 s (std., 1.290) and the classification took 0.106 s (std., 0.028) using a standard laptop with an Intel Core i7 (10th generation) without a GPU.

## 4. Discussion

We validated the chronic use of EEG signals in a real-life authentication setting. Every participant achieved an accuracy of at least 81.3%, with an average accuracy of 90% across the participants. These accuracies were lower than the ones achieved in previous studies using datasets from an acute recording session and using clinical-grade EEG setups. However, we showed that chronic EEG-based authentication using a commercial-grade dry-electrode headset is not infeasible. Furthermore, we were using rather simple temporo-spectral features and a simple bagging technique to improve the accuracy of the LSTM network. The bagging approach also achieved around 0.75 accuracy when the training set only contained 12 data points, indicating good scalability. Interestingly, the accuracies further improved when combining data from the left and right performed/imagined motor tasks (Table 1), but the FAR decreased whilst the FRR increased. Note that the FAR and FRR can be adjusted by introducing or removing tasks. Based on the Wilcoxon signed-rank test results, we conclude that a single-task setup usually achieves a higher FAR and lower accuracy and FRR than a two-task setup, indicating that the former has a higher probability of accepting an imposter and a lower probability of rejecting an authorized user. This provides flexibility to adjust the authentication system based on the desired application. For private and confidential application systems, such as accessing a bank account, specific tasks could be combined to guarantee no false acceptance at all, while, for applications that have low-security concerns but high time costs, such as an automatic check-in system in a hotel, a simple single-task scheme might be optimal. The classification network also allows for a retraining of the network with subsequent authentication attempts, once the data have been labeled correctly, without a heavy calculation requirement. When further comparing the results obtained in Figure 8 and Figure 11, we found that the mean and median values were similar, yet the latter had much larger variance across participants, especially for the single-task authentication scheme, indicating that the authentication system performed worse in rejecting imposters. However, the two-task authentication scheme still showed good performance, with a minimum accuracy of 0.83 and maximum accuracy of 0.99.

In future work, we plan to investigate the following.

1. Specialized decoders/features: Unlike [13], where the LSTM worked with a CNN, or [23], where advanced data handling methods such as cross-correlation and transient removal were applied to achieve better-quality temporal features, we used only basic techniques. It could also be interesting to compare different decoders and features and to investigate which one is most suited for real-life chronic EEG authentication.
2. Electrode/sub-band selection: We used eight electrodes and five sub-bands, but, when eliminating some electrodes or sub-bands, the results improved. Moreover, resting-state signals could be adopted to guide the optimization, as shown in [13], where eye blinking was used, albeit for user identification.
3. Ensemble techniques: Although plausible, the proposed bagging with only one base classifier could be improved. According to Figure 7, no significant improvement was found when further increasing the ensemble size M. This may be caused by either insufficient training data or the limited decoding power of the LSTM-based model. Therefore, collecting more data or introducing other ensemble techniques could be investigated, for example, the mix-boost ensemble method proposed in [8], where better performance could be achieved when using multiple types of base classifiers rather than by varying hyperparameters for a single type of base classifier.

Another issue with the proposed ensemble method is the training time. Compared to [7], our total time was significantly lower—only 144 s to train a model (consisting of nine bagged LSTMs) using a standard laptop with an Intel Core i7 (10th generation) without a GPU—and, when training large ensembles, the training time increased linearly. Whilst we realize that the subject population was limited, we wanted to emphasize the importance of design choices when it comes to a practical authentication system and the difficulties of using EEG for such a task.

## 5. Conclusions

We validated, for the first time, the chronic use of a brainwave authentication decoder using a commercial dry-electrode EEG headset in a real-world setting. Data were collected outside the lab for most of the participants, and multiple recordings over different days were performed on the same participant. An accuracy of 90% was achieved by using a bagging technique combined with a rather shallow standard LSTM network applied to simple spectrogram-based features, instead of advanced classification methods and signal features. The proposed bagging approach showed good scalability, yielding an accuracy around 75% with only 12 data points. Moreover, thanks to the simplicity of the decoder structure, the loading, training, and classification times were around 0.135 s (loading nine LSTM-based ensembles), 16.59 s (training one LSTM-based ensemble), and 0.106 s (classification from nine LSTM-based ensembles). We recommend that, to suit different application scenarios, multiple tasks should be combined. The more tasks the user needs to successfully complete, the lower the FAR and the higher the FRR. Given our results on chronic usage in a real-world setting, we believe that EEG-based authentication is feasible and that performance can be further improved by expanding the population base, by incorporating more advanced signal features and decoders, and by investigating the impact of different design options on the authentication paradigm.

## Figures and Tables

**Table 1:** Results averaged over subjects and folds of the left or right arm and combined motor tasks.

Scheme	Left Performed Motor	Right Performed Motor	Combined Performed Motor	Left Imagined Motor	Right Imagined Motor	Combined Imagined Motor	Combined Task
Averaged Acc.	0.884	0.887	0.926	0.883	0.888	0.925	0.930
Averaged FAR	0.077	0.073	0.025	0.080	0.072	0.026	0.019
Averaged FRR	0.039	0.040	0.050	0.038	0.041	0.049	0.051

## Data Availability

The data are not publicly available but can be made available upon request.

## Supplementary Materials

The following is available online: https://www.mdpi.com/article/10.3390/bios11100404/s1. Table S1 “Average results of accuracies per subject from 5-fold cross-validation, reported in the form of: mean (std)”; Table S2 “Average results of FAR per subject from 5-fold cross-validation, reported in the form of: mean (std)”; Table S3 “Average results of FRR per subject from 5-fold cross-validation, reported in the form of: mean (std)”; Table S4 “Wilcoxon signed-rank test results for pair-wise paradigm comparison of accuracies: marked in bold when statistically significant (α=0.05)”; Table S5 “Wilcoxon signed-rank test results for pair-wise paradigm comparison of FARs: marked in bold when statistically significant (α=0.05)”; Table S6 “Wilcoxon signed-rank test results for pair-wise paradigm comparison of FRRs: marked in bold when statistically significant (α=0.05)”.
