# Supplementary material for: Chronic Study on Brainwave Authentication in a Real-Life Setting: An LSTM-Based Bagging Approach
Source: Biosensors (Basel). 2021 Oct 18;11(10):404. doi: 10.3390/bios11100404 (PMC8533875; doi:10.3390/bios11100404)
Supplement: Supplementary file 1 [file biosensors-11-00404-s001.zip › biosensors-1332051-supplementary.pdf]

biosensors

MDPI

Table S1. Average results of accuracies per subject from 5-fold cross-validation, reported in the form of: mean (std).

Subject	Left perf.motor	Right perf.motor	Combined perf.motor	Left imag.motor	Right imag.motor	Combined imag.motor	Combined task
1	0.893 (0.028)	0.867 (0.030)	0.926 (0.006)	0.889 (0.032)	0.888 (0.033)	0.928 (0.009)	0.930 (0.005)
2	0.852 (0.041)	0.838 (0.066)	0.897 (0.037)	0.865 (0.029)	0.822 (0.086)	0.893 (0.037)	0.904 (0.031)
3	0.906 (0.046)	0.880 (0.030)	0.931 (0.030)	0.893 (0.048)	0.885 (0.043)	0.927 (0.031)	0.934 (0.023)
4	0.946 (0.017)	0.951 (0.015)	0.954 (0.013)	0.946 (0.017)	0.944 (0.015)	0.953 (0.012)	0.953 (0.010)
5	0.916 (0.020)	0.922 (0.021)	0.950 (0.006)	0.902 (0.018)	0.917 (0.030)	0.944 (0.017)	0.950 (0.007)
6	0.949 (0.030)	0.944 (0.027)	0.964 (0.015)	0.947 (0.028)	0.938 (0.021)	0.962 (0.016)	0.966 (0.015)
7	0.913 (0.018)	0.885 (0.046)	0.954 (0.020)	0.913 (0.027)	0.879 (0.056)	0.951 (0.018)	0.955 (0.021)
8	0.838 (0.048)	0.861 (0.074)	0.904 (0.030)	0.850 (0.022)	0.886 (0.058)	0.919 (0.013)	0.920 (0.011)
9	0.904 (0.016)	0.926 (0.032)	0.935 (0.029)	0.894 (0.007)	0.929 (0.036)	0.939 (0.026)	0.936 (0.030)
10	0.891 (0.034)	0.871 (0.044)	0.913 (0.034)	0.892 (0.030)	0.888 (0.046)	0.915 (0.029)	0.917 (0.034)
11	0.884 (0.042)	0.883 (0.053)	0.936 (0.025)	0.877 (0.044)	0.893 (0.048)	0.938 (0.025)	0.940 (0.027)
12	0.893 (0.051)	0.867 (0.020)	0.926 (0.012)	0.889 (0.051)	0.888 (0.021)	0.928 (0.015)	0.930 (0.008)
13	0.852 (0.042)	0.838 (0.055)	0.897 (0.027)	0.865 (0.036)	0.822 (0.065)	0.893 (0.044)	0.904 (0.025)
14	0.906 (0.060)	0.880 (0.028)	0.931 (0.018)	0.893 (0.045)	0.885 (0.036)	0.927 (0.014)	0.934 (0.009)
15	0.946 (0.053)	0.951 (0.050)	0.954 (0.031)	0.946 (0.064)	0.944 (0.053)	0.953 (0.036)	0.953 (0.028)
Mean	0.884 (0.041)	0.887 (0.034)	0.926 (0.024)	0.883 (0.037)	0.888 (0.036)	0.925 (0.026)	0.930 (0.021)

Table S2. Average results of FAR per subject from 5-fold cross-validation, reported in the form of: mean (std).

Subject	Left perf.motor	Right perf.motor	Combined perf.motor	Left imag.motor	Right imag.motor	Combined imag.motor	Combined task
1	0.051 (0.030)	0.080 (0.030)	0.010 (0.006)	0.053 (0.036)	0.051 (0.032)	0.006 (0.007)	0.004 (0.004)
2	0.107 (0.036)	0.104 (0.056)	0.041 (0.033)	0.093 (0.025)	0.117 (0.084)	0.042 (0.036)	0.030 (0.029)
3	0.077 (0.047)	0.096 (0.029)	0.036 (0.023)	0.087 (0.048)	0.092 (0.044)	0.038 (0.026)	0.028 (0.020)
4	0.021 (0.018)	0.013 (0.011)	0.008 (0.007)	0.019 (0.014)	0.024 (0.019)	0.012 (0.007)	0.008 (0.006)
5	0.062 (0.028)	0.057 (0.031)	0.021 (0.020)	0.075 (0.031)	0.064 (0.037)	0.027 (0.029)	0.020 (0.018)
6	0.041 (0.037)	0.049 (0.030)	0.023 (0.021)	0.041 (0.036)	0.053 (0.026)	0.025 (0.022)	0.020 (0.020)
7	0.062 (0.034)	0.082 (0.045)	0.010 (0.007)	0.066 (0.042)	0.086 (0.070)	0.013 (0.008)	0.008 (0.006)
8	0.100 (0.052)	0.078 (0.072)	0.025 (0.032)	0.090 (0.019)	0.052 (0.051)	0.011 (0.012)	0.009 (0.011)
9	0.062 (0.023)	0.046 (0.018)	0.021 (0.016)	0.072 (0.030)	0.043 (0.029)	0.018 (0.017)	0.018 (0.016)
10	0.069 (0.027)	0.101 (0.043)	0.040 (0.030)	0.074 (0.028)	0.078 (0.053)	0.038 (0.030)	0.033 (0.029)
11	0.077 (0.049)	0.079 (0.033)	0.022 (0.010)	0.088 (0.060)	0.071 (0041)	0.023 (0.015)	0.018 (0.010)
12	0.130 (0.062)	0.056 (0.019)	0.018 (0.009)	0.114 (0.065)	0.055 (0.030)	0.011 (0.015)	0.008 (0.006)
13	0.101 (0.050)	0.088 (0.067)	0.038 (0.031)	0.127 (0.043)	0.128 (0.080)	0.068 (0.051)	0.035 (0.027)
14	0.063 (0.060)	0.085 (0.038)	0.014 (0.019)	0.076 (0.043)	0.092 (0.039)	0.017 (0.011)	0.008 (0.010)
15	0.136 (0.047)	0.079 (0.049)	0.040 (0.033)	0.119 (0.053)	0.068 (0.053)	0.039 (0.037)	0.030 (0.031)
Mean	0.077 (0.032)	0.073 (0.024)	0.025 (0.012)	0.080 (0.029)	0.072 (0.028)	0.026 (0.017)	0.019 (0.011)

Table S3. Average results of FRR per subject from 5-fold cross-validation, reported in the form of: mean (std).

**Subject**	Left perf.motor	Right perf.motor	Combined perf.motor	Left imag.motor	Right imag.motor	Combined imag.motor	Combined task
1	0.056 (0.007)	0.053 (0.007)	0.064 (0.002)	0.058 (0.009)	0.062 (0.008)	0.066 (0.002)	0.066 (0.002)
2	0.041 (0.013)	0.057 (0.013)	0.062 (0.009)	0.042 (0.009)	0.061 (0.002)	0.065 (0.002)	0.065 (0.003)
3	0.017 (0.006)	0.023 (0.011)	0.033 (0.010)	0.021 (0.010)	0.023 (0.012)	0.036 (0.011)	0.038 (0.011)
4	0.034 (0.018)	0.036 (0.013)	0.038 (0.011)	0.035 (0.016)	0.032 (0.016)	0.036 (0.014)	0.038 (0.010)
5	0.022 (0.013)	0.021 (0.017)	0.029 (0.015)	0.023 (0.015)	0.019 (0.015)	0.029 (0.014)	0.031 (0.014)
6	0.010 (0.011)	0.007 (0.005)	0.013 (0.011)	0.012 (0.012)	0.009 (0.007)	0.013 (0.013)	0.013 (0.013)
7	0.025 (0.017)	0.033 (0.020)	0.037 (0.018)	0.021 (0.016)	0.035 (0.022)	0.036 (0.021)	0.037 (0.019)
8	0.063 (0.011)	0.062 (0.009)	0.071 (0.002)	0.060 (0.009)	0.063 (0.010)	0.071 (0.002)	0.071 (0.001)
9	0.035 (0.023)	0.029 (0.020)	0.045 (0.024)	0.034 (0.024)	0.029 (0.021)	0.043 (0.024)	0.046 (0.023)
10	0.040 (0.019)	0.029 (0.011)	0.047 (0.013)	0.034 (0.019)	0.035 (0.013)	0.047 (0.010)	0.050 (0.011)
11	0.039 (0.035)	0.038 (0.034)	0.042 (0.035)	0.036 (0.034)	0.036 (0.033)	0.038 (0.036)	0.042 (0.033)
12	0.053 (0.017)	0.056 (0.012)	0.068 (0.007)	0.053 (0.017)	0.059 (0.014)	0.069 (0.007)	0.069 (0.005)
13	0.068 (0.009)	0.064 (0.014)	0.073 (0.006)	0.060 (0.013)	0.055 (0.017)	0.070 (0.007)	0.073 (0.002)
14	0.055 (0.006)	0.033 (0.014)	0.056 (0.007)	0.048 (0.011)	0.033 (0.012)	0.052 (0.008)	0.059 (0.003)
15	0.027 (0.010)	0.063 (0.005)	0.066 (0.004)	0.030 (0.019)	0.063 (0.000)	0.068 (0.006)	0.069 (0.004)
Mean	0.039 (0.017)	0.040 (0.018)	0.050 (0.018)	0.038 (0.015)	0.041 (0.018)	0.049 (0.018)	0.051 (0.018)

Table S4. Wilcoxon signed-rank test results for pair-wise paradigm comparison of accuracies: marked in bold when statistically significant ($\alpha = 0.05$).

**Paradigms**	Left perf.motor	Right perf.motor	Combined perf.motor	Left imag.motor	Right imag.motor	Combined imag.motor	Combined task
Left perf.motor		0.96387	**0.00006**	0.55994	0.93408	**0.00006**	**0.00006**
Right perf.motor			**0.00006**	0.58972	0.87897	**0.00006**	**0.00006**
Combined perf.motor				**0.00006**	**0.00006**	0.85449	**0.00031**
Left imag.motor					0.5614	**0.00006**	**0.00006**
Right imag.motor						**0.00006**	**0.00006**
Combined imag.motor							**0.0014**
Combined task							

Table S5. Wilcoxon signed-rank test results for pair-wise paradigm comparison of FARs: marked in bold when statistically significant (α = 0.05).

**Paradigms**	Left perf.motor	Right perf.motor	Combined perf.motor	Left imag.motor	Right imag.motor	Combined imag.motor	Combined task
Left perf.motor		0.94611	**0.00006**	0.5719	0.9032	**0.00006**	**0.00006**
Right perf.motor			**0.00006**	0.47913	0.85883	**0.00006**	**0.00006**
Combined perf.motor				**0.00006**	**0.00006**	0.71124	**0.00012**
Left imag.motor					0.57074	**0.00006**	**0.00006**
Right imag.motor						**0.00006**	**0.00006**
Combined imag.motor							**0.00012**
Combined task							

Table S6. Wilcoxon signed-rank test results for pair-wise paradigm comparison of FRRs: marked in bold when statistically significant (α = 0.05).

**Paradigms**	Left perf.motor	Right perf.motor	Combined perf.motor	Left imag.motor	Right imag.motor	Combined imag.motor	Combined task
Left perf.motor		0.96637	**0.00006**	0.36353	0.77368	**0.00067**	**0.00006**
Right perf.motor			**0.00006**	0.79187	0.5957	**0.00024**	**0.00006**
Combined perf.motor				**0.00006**	**0.00006**	0.59033	**0.00018**
Left imag.motor					0.57166	**0.00006**	**0.00006**
Right imag.motor						**0.00006**	**0.00006**
Combined imag.motor							**0.00037**
Combined task							
